# Hepatic COX-2 expression protects mice from an alcohol-high fat diet-induced metabolic disorder by involving protein acetylation related energy metabolism

**DOI:** 10.1016/j.alcohol.2020.08.007

**Published:** 2021-03-02

**Authors:** Minjie Chen, Xicui Sun, Wei Wei, Carme Cucarella, Paloma Martín-Sanz, Marta Casado, Liya Pi, Bin Ren, Qi Cao

**Affiliations:** a Diagnostic Radiology & Nuclear Medicine, University of Maryland School of Medicine, Baltimore, MD, USA; b Instituto de Biomedicina de Valencia, IBV-CSIC, Jaume Roig 11, Valencia, 46010, Spain; c Instituto de Investigaciones Biomédicas (IIB) “Alberto Sols”, CSIC-UAM, Arturo Duperier 4, Madrid, 28029, Spain; d Centro de Investigación Biomédica en Red de Enfermedades Hepáticas y Digestivas (CIBERehd), Monforte de Lemos 3-5, Madrid, 28029, Spain; e Department of Pediatrics, College of Medicine, Gainesville, FL, USA; f Department of Surgery, University of Alabama at Birmingham, Birmingham, AL, USA

**Keywords:** COX-2, fatty liver disease, metabolism, protein acetylation

## Abstract

**Purpose:**

A diet high in fat and ethanol often results in chronic metabolic disorder, hepatic steatosis, and liver inflammation. Constitutive hepatic cyclooxygenase-2 (COX-2) expression could protect from high fat-induced metabolism disturbance in a murine model. In this study, we explored the influence of hCOX-2 transgenic [TG] to high fat with ethanol-induced metabolic disorder and liver injury using a mouse animal model.

**Methods:**

12-week-old male hepatic hCOX-2 transgenic (TG) or wild type mice (WT) were fed either a high fat and ethanol liquid diet (HF+Eth) or a regular control diet (RCD) for 5 weeks (four groups: RCD/WT, RCD/TG; HF+Eth/TG, HF+Eth/WT). We assessed metabolic biomarkers, cytokine profiles, histomorphology, and gene expression to study the impact of persistent hepatic COX-2 expression on diet-induced liver injury.

**Results:**

In the HF+Eth diet, constitutively hepatic human COX-2 expression protects mice from body weight gain and white adipose tissue accumulation, accompanied by improved IPGTT response, serum triglyceride/cholesterol levels, and lower levels of serum and liver inflammatory cytokines. Histologically, hCOX-2 mice showed decreased hepatic lipid droplets accumulation, decreased hepatocyte ballooning, and improved steatosis scores. Hepatic hCOX-2 overexpression enhanced AKT insulin signaling and increased fatty acid synthesis in both RCD and HF+Eth diet groups. The anti-lipogenic effect of hCOX-2 TG in the HF+Eth diet animals was mediated by increasing lipid disposal through enhanced β-oxidation via elevations in the expression of PPARa and PPARg, and increased hepatic autophagy as assessed by the ratio of autophagy markers LC3 II/I in hepatic tissue. Various protein acetylation pathway components, including HAT, HDAC1, SIRT1, and SNAIL1, were modulated in hCOX-2 TG mice in either RCD or HF+Eth diet.

**Conclusions:**

Hepatic human COX-2 expression protected mice from the metabolic disorder and liver injury induced by a high fat and ethanol diet by enhancing hepatic lipid expenditure. Epigenetic reprogramming of diverse metabolic genes might be involved in the anti-lipogenic effect of COX-2.

## Background

Epidemiologically, a high fat diet and alcohol consumption are two leading causes of liver damage and metabolic disturbance. The influences of ethanol on a high fat diet depend on the stage of liver injury and the amount of alcohol consumption (Paulson, Hong, Holcomb, & Nunez, 2010; Srinivasan, Shawky, Kaphalia, Thangaraju, & Segar, 2019; Traversy & Chaput, 2015). In animal models, it has been demonstrated that ethanol exaggerates liver inflammation, while conversely it has been shown that, according to the frequency of consumption, ethanol could partially protect mice/rats from high fat diet-induced insulin resistance and obesity (Chang et al., 2015;Coker et al., 2020;Demori, Voci, Fugassa, & Burlando, 2006;Feng et al., 2012;Lindtner et al., 2013;Wang, Seitz, & Wang, 2010).

Emerging evidence suggests a critical role for cyclooxygenase-2 (COX-2) in the link between inflammation and nutrition metabolism. Usually, COX-2 is induced in acute infection in liver Kupffer cells, but it is recognized in chronic viral infection as well; both conditions are accompanied by weight loss (Casado et al., 2007; Chen et al., 2015;Cheng et al., 2004; Francés et al., 2013, 2015). Recently, it has been reported that hepatic COX-2 transgenic mice are protected from streptozotocin (STZ) or high fat-induced hyperglycemia, insulin resistance, and liver damage (Carboneau, Breyer, & Gannon, 2017; Motiño et al., 2019). It has been well documented that COX-2 TG mice are protected from HF-induced hepatic steatosis, weight gain, and insulin resistance, but ethanol’s effect on a high fat diet in nutrition metabolism and liver injury has not been explored.

Researchers have described epigenetic modification in modulating metabolic gene expression under nutritional stress (Choi & Friso, 2010). Acetylation of histones by ethanol consumption or a high fat diet in animal models is established (Aroor, Restrepo, Kharbanda, & Shukla, 2014; Carrer et al., 2017; Meyer et al., 2018; Shepard & Tuma, 2009; Shukla, Aroor, Restrepo, Kharbanda, & Ibdah, 2015). Mechanically, the regulation of energy metabolism by NAD-dependent deacetylase sirtuin-1 (SIRT1) (Liang, Kume, & Koya, 2009; Salminen, Kauppinen, & Kaarniranta, 2016), and recently published studies showing that the insulin/SNAIL1 axis epigenetically suppresses lipogenesis, offer a novel theory to explain the chromatin remodeling that occurs as a result of challenges presented by a high fat diet (Liu et al., 2018; Rowe et al., 2011; Sun et al., 2016). We theorize that protein acetylation is one underlying mechanism that regulates metabolism-related gene expression epigenetically, and further mediates the protective function of hepatic COX-2 overexpression.

We have investigated fat metabolism in COX-2 TG or wild type (WT) mice under a high fat with ethanol diet, revealing that COX-2 TG mice are protected from diet-induced weight gain, metabolic syndrome, and liver damage. Furthermore, our results provide insight into nutritional reprogramming through histone and protein acetylation under the influence of hepatic COX-2 overexpression in the animal model.

## Materials and methods

### Animals and diets

The University of Maryland, Baltimore (UMB) is an Association for Assessment and Accreditation of Laboratory Animal Care (AAALAC)-accredited institution. All procedures were approved by the Institutional Animal Care and Use Committee at UMB. COX-2 TG mice, which constitutively express human COX-2 in hepatocytes under the control of the ApoE promoter and its specific hepatic control region (Casado et al., 2007), and their corresponding WT littermates, were fed with the Lieber DeCarli high fat with ethanol diet (HF+Eth) or a regular control diet (RCD). 12-week-old male mice were acclimated for RCD containing 18% of calories as protein, 35% as fat, and 47% as carbohydrates (Lieber-Decarli’82 Shake and Pour control liquid diet, Bio-Serv, product no. F1259SP) for 1 week. The animals were then divided into two dietary groups: a) control diet (RCD) or b) HF+Eth diet as 27.5% of calories from ethanol and 40% of calories from corn oil (Dyets, product no. 712031 with corn oil 401150) for 5 weeks. The HF+Eth diet group was adapted gradually to a 5% (v/v) ethanol diet over 1 week (2 days × 1.25% ethanol, 2 days × 2.5% ethanol). Mice weights were recorded every week from 12 weeks until 18 weeks, and blood was collected at euthanization.

### IPGTT and ITT

After 5 weeks on the RCD or HF+Eth diet, mice underwent a glucose tolerance test (IPGTT) and insulin tolerance test (ITT). For the GTT, mice fasted for 16 hours were intraperitoneally (i.p.) injected with glucose (2 g/kg body weight), and tail vein blood was taken at 0, 15, 30, 60, and 120 minutes. Glucose levels were determined using an automatic glucometer (Contour, Bayer). For the ITT, mice fasted for 4 hours and then were i.p. injected with insulin (0.75 IU/kg body weight). Blood glucose was measured from tail vein blood at 0,15, 30, 60, and 120 minutes as described above.

### Histological analysis of liver and peritoneal adipose tissue

Tissue was fixed in 4% paraformaldehyde (PFA), embedded in paraffin, cut into 5-μm sections, and subjected to H&E staining. Slides were scored for steatosis and ballooning according to the non-alcoholic fatty liver disease (NAFLD) activity score (NAS) (Brunt et al., 2011; Kleiner et al., 2005).The liver was snap-frozen in Optimal cutting temperature compound (OCT) and cryostat into 10-μm sections. After a brief fixation in 4% PFA, slides were stained with freshly made Oil Red O and counterstained with hematoxylin. The image was acquired and analyzed by calculating the red area to total area (%) using ImageJ software. Sirus Red/Fast Green staining was performed on paraffin slides according to the manufacturer’s instruction (Sirus Red/Fast Green Collagen Staining Kit, Chondrex) and results were analyzed by Image J as well. The analysis was performed on three successive sections per slide, and at least nine sections from three non-consecutive slides per mouse were examined. All analyses were performed by an investigator blinded to group assignment.

### Plasma cytokine analysis

To rapidly harvest blood samples for cytokine analysis, mouse retro-orbital blood samples were collected and subjected to centrifugation for 15 minutes at 2000×g using a refrigerated centrifuge. The supernatants (serum) were then transferred to new tubes and stored at −80 °C. Plasma cytokine levels were assessed using LEG-ENDplex (BioLegend; San Diego, California, United States) as per manufacturer’s instructions. Briefly, 25 μΐ of mouse serum was incubated with cytokine-labeled beads, and then the signal was acquired by flow cytometry and quantified by LEGENDplex software.

### Plasma and liver AST, TG, cholesterol, and insulin measurement

Plasma insulin measurement was performed according to manufacturer’s instructions (Ultra-Sensitive Mouse Insulin ELISA Kit, Crystal Chemical; Elk Grove Village, Illinois, United States). Plasma AST, plasma and liver cholesterol, and triglyceride levels using commercially available kits (BioVision Inc.; Milpitas, California, United States) were determined according to our published methods (Cao, Batey, Pang, & Clancy, 1999; Lieber et al., 2004). Briefly, triglycerides from serum and liver tissue lysate were converted into triglycerol and fatty acid and detected by a triglyceride probe at Ex/Em = 535/590 nm, according to the manufacturer’s instructions.

### Real-time polymerase chain reactions

Mouse livers were snap-frozen in liquid nitrogen and kept at −80 °C. Total RNA from liver was extracted and purified using the Trizol reagent (Invitrogen, Thermo Fisher Scientific; Carlsbad, California, United States). The quality of RNA was assessed by determining A_280_:A_260_ as assessed by Nano-drop technology. Two mg of total DNase-treated RNA was reverse-transcribed into cDNA using High Capacity cDNA Reverse Transcription Kits (Applied Biosystems, Thermo Fisher Scientific; Foster City, California) as per manufacturer’s instructions. Real-time PCR was performed using LightCycler® 480 SYBR Green I Master in the LightCycler® (Roche Life Science; Indianapolis, Indiana, United States). Reactions were performed in a total volume of 10 μL containing 1 ng cDNA, 0.2 μM of each primer, and 5 μL of the SYBR® Green reaction mix. The amplification protocol was as follows: 95 °C for 5 minutes, 45 cycles of 95 °C for 10 seconds, 60 °C for 20 seconds, and 72 °C for 30 seconds. Following amplification, a dissociation curve analysis was performed to ensure the purity of the PCR product. The specific sense and antisense primers are listed in Table 1. In each sample, 2ΔΔ^Ct^ was calculated and used to represent its gene expression level.

### Western blot

Standard Western blot techniques were performed. Briefly, 50—200 mg protein from liver tissue lysate in RIPA buffer with proteinase inhibitor were loaded. Antibodies used are listed in Table 2. Signals were detected by chemiluminescence and analyzed by ImageJ.

### Statistics

Statistics were performed using GraphPad Prism (GraphPad Software; San Diego, California). The difference between the two groups was analyzed by the two-tailed Student’s *t* test. IPGTT, ITT, and growth curves were analyzed by one-way ANOVA. *p* < 0.05 was considered statistically significant.

## Results

### COX-2-TG mice were protected from HF+Eth-induced weight gain, adipose tissue deposition, and hyperlipidemia

To explore the effects of hepatic COX-2 expression under nutritional stress, we exposed COX-2 TG or WT mice to control diet or high fat diet with ethanol diet for 5 weeks. Since ethanol’s effect on a high fat diet in nutrition metabolism and liver injury has not been explored in COX-2 TG mice, we wanted to further elucidate the influence of ethanol in 5 weeks’ exposure time of HF+Eth diet in COX-2 TG mice on weight gain, adipose tissue accumulation, metabolic panel, and inflammatory status.

**Fig. 1.**
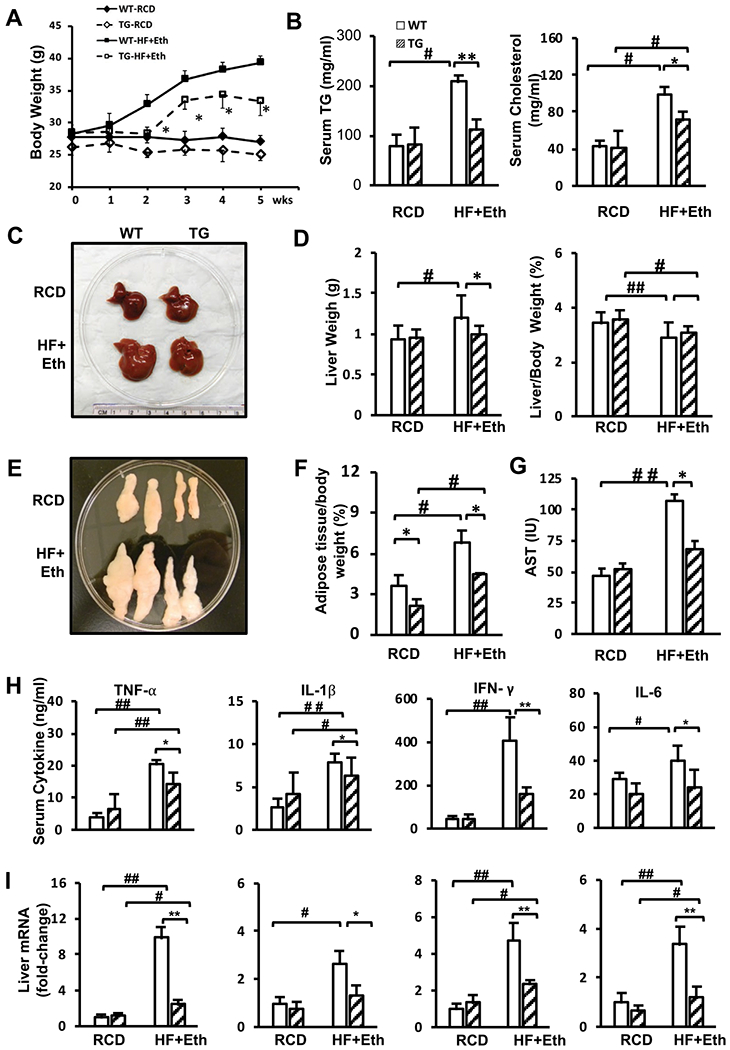
Cox-2-TG mice were protected from HF+Eth-induced weight gain, adipose tissue deposition, and hyperlipidemia. **(A)** Weekly weight change after WT and TG mice were fed with a liquid control diet or a high fat + ethanol diet for 5 weeks. **(B)** Levels of serum triglyceride and cholesterol after mice were fed with RCD or HF+Eth diet. **(C)** Representative pictures of livers from WT and TG mice on RCD or HF+Eth diet after 5 weeks. **(D)** Liver weight and relative liver mass to body weight. **(E)** Representative pictures of epididymal white adipose tissue (eWAT) from WT and TG mice on RCD or HF+Eth diet after 5 weeks. **(F)** Weight of eWAT adjusted to body weight. **(G)** Serum AST level. **(H)** Serum cytokine levels of TNF-α, IL-1β, IFN-γ, and IL-6 in WT and TG mice on RCD or HF+Eth diet for 5 weeks measured by LegendPlex. **(I)** Liver cytokine mRNA expression levels of *TNF-α*, *Il-1β*, *INF-γ*, and *Il-6* genes in WT and TG mice on RCD or HF+Eth diet for 5 weeks. N = 6—8 mice in each group, data are presented as mean ± SD. *p < 0.05, **p < 0.01 WT vs. HF+Eth; #*p* < 0.05, ##*p* < 0.01 RCD vs. HF+Eth.

During HF+Eth feeding, COX-2 TG mice started to gain weight after 3 weeks, as opposed to the quick onset of weight gain in WT mice from the first week. At the end of 5 weeks of feeding, the average weight of the COX-2 TG mice was 33.4 ± 1.1 g, which was 15% lower than the weight gain seen in the WT mice on the HF+Eth diet. In the regular control diet, there was no statistical difference in body weight between the TG and WT mice (Fig. 1A). The liver weight increased from 0.94 ± 0.16 g to 1.19 ± 0.29 g in the WT HF+Eth diet group (Fig. 1C and D), and liver-to-body weight ratio was relatively decreased (Fig. 1D), partially attributable to accumulated white adipose tissue on the HF+Eth diet (Fig. 1E and F). In HF+Eth diet mice, hepatomegaly was reduced by 18% in TG mice compared to WT mice, and white adipose tissue accumulation was greatly decreased from 6.79 ± 0.09% to 4.45 ± 0.11%, respectively (Fig. 1E and F). Serum AST level showed that HF + Eth diet could greatly impair the liver function, while the liver injury was absent in the COX-2TG mice with HF+Eth diet (Fig. 1G).

To evaluate the impact of nutritional stress from the HF+Eth diet, we analyzed the metabolic panel of TG and WT mice serum on control and HF+Eth diets for 5 weeks. Both triglycerol and cholesterol levels were greatly increased in WT mice on the HF + Eth diet, but this increase was significantly less in the TG mice on the same diet (Fig. 1B). These results indicate that COX-2 TG may protect mice from an HF+Eth diet-induced weight gain, adipose tissue deposition, and hyperlipidemia.

Cytokines secreted by the liver lead to systemic inflammation, which is the link between NFALD and obesity (Adams, Anstee, Tilg, & Targher, 2017; Ballestri et al., 2016; Sarwar, Pierce, & Koppe, 2018). Previous work indicated that low levels of IFN-γ and TNF-α are a critical physiological factor that protects COX-2 TG mice from obesity induced by a high fat diet (Francés et al., 2015; Motiño et al., 2016). In our study, HF+Eth diet upregulated cytokines in WT mice, whereas serum cytokines of TG mice exhibited a significantly decreased pattern relative to the WT mice (Fig. 1H). We found that liver mRNA levels of cytokines from COX-2 TG mice were significantly decreased compared to the WT mice in the HF+Eth diet (Fig. 1I). Thus, in the mice fed the HF+Eth diet, suppressed cytokine levels contributed to the protective effect from COX-2 hepatic overexpression.

### COX-2-TG mice were protected from HF+Eth-induced glucose intolerance and insulin resistance

**Fig. 2.**
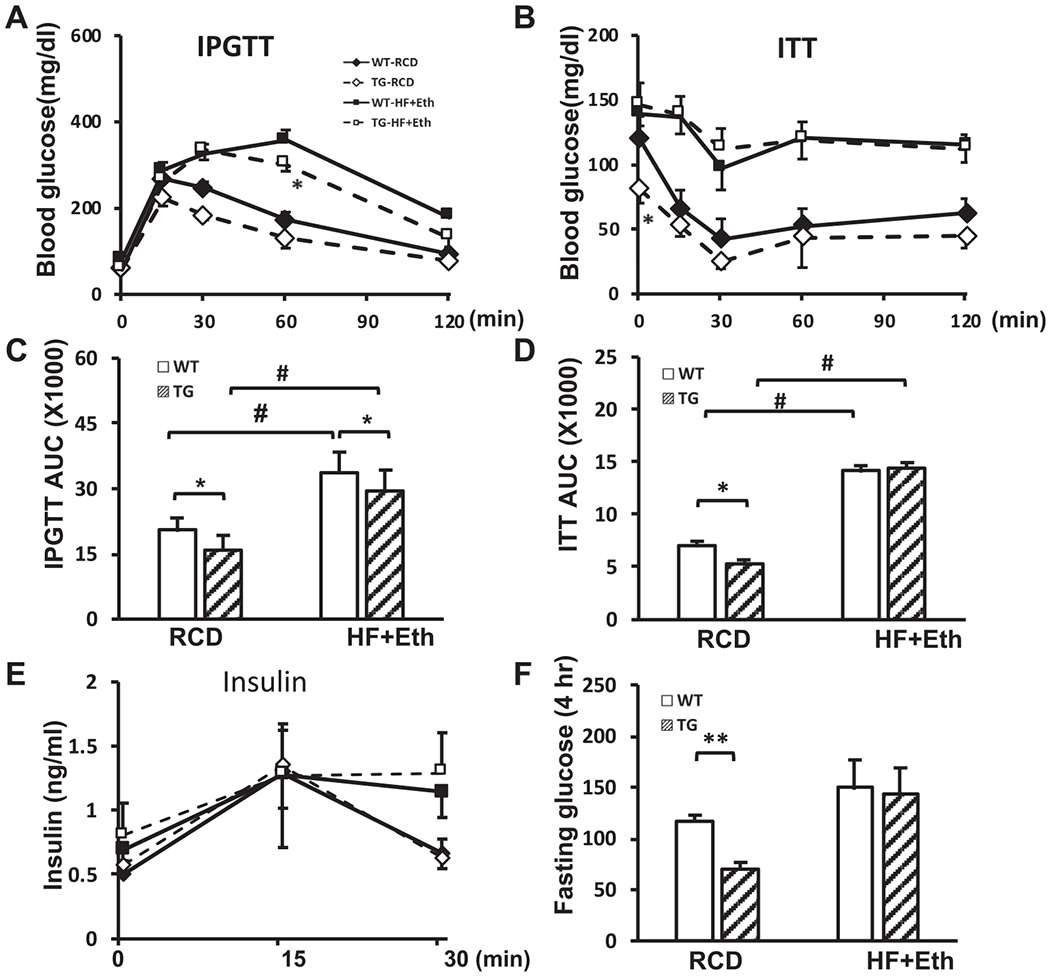
Cox-2 expression protects against HF+Eth-induced glucose intolerance and insulin resistance. **(A)** IPGTT analysis in WT and TG mice on RCD or HF+Eth diet for 4 weeks. 2 g/kg glucose were i.p. injected after 16 hours fasting. Mice plasma glucose level was checked at 0, 30, 15, 60, 90, and 120 minutes. **(B)** ITT analysis in WT and TG mice on RCD or HF+Eth diet for 4 weeks. 0.75 IU/kg insulin was i.p. injected after 4 hours fasting. Plasma glucose level was checked at 0, 30, 15, 60, 90, and 120 minutes. **(C)** Calculated area under curve (AUC) after IPGTT. **(D)** Calculated AUC after ITT. **(E)** Serum insulin levels during IPGTT challenge. **(F)** Plasma glucose levels after 4 hours fasting as presented in **(B)** ITT. N = 6—8 mice in each group, data are presented as mean ± SD. *p < 0.05, **p < 0.01 WT vs. HF + Eth; #*p* < 0.05, ##*p* < 0.01 RCD vs. HF+Eth.

Weight gain resulting from a high fat diet is closely associated with glucose intolerance and insulin resistance, but conflicting results exist regarding the effects of alcohol on the insulin response and diabetes (Traversy & Chaput, 2015; Wang et al., 2010; Zhong & Lemasters, 2018). We performed IPGTT and ITT to analyze glucose homeostasis in TG and WT mice under both the regular control diet and the HF+Eth diet. After overnight fasting (16 hours), there was no difference in blood glucose levels between groups. After the glucose challenge, WT mice on the HF+Eth diet had significantly increased blood glucose levels (Fig. 2A and 2B). We observed partially restored glucose sensitivity in TG mice fed the HF+Eth diet compared to WT mice, with slightly but significantly suppressed area under curve (AUC) values (Fig. 2C and 2D). Both WT and TG mice showed escalated and sustained insulin levels similar to the mice fed the HF+Eth diet, indicating comparable pancreatic islet function in response to the high fat with ethanol diet (Fig. 2E).

Next, we evaluated the insulin sensitivity by ITT. Consistent with the IPGTT result, WT mice exhibited impaired glucose clearance on the HF+Eth diet, but in contrast, there was no statistically significant difference of the AUC between TG and WT on the HF+Eth diet (Fig. 2B and 2D). Surprisingly, the fasting glucose (4 hours) levels in the ITT were lower in TG mice than WT mice on the control diet, as well as the AUC of TG and WT mice (Fig. 2F). These results suggest either enhanced insulin response or decreased glycogen storage in TG mice on the regular control diet, but this difference was moderated in COX-2 TG mice on the HF+Eth diet.

### COX-2 TG mice were protected from HF+Eth-induced liver steatosis and adipose tissue ballooning

**Fig. 3.**
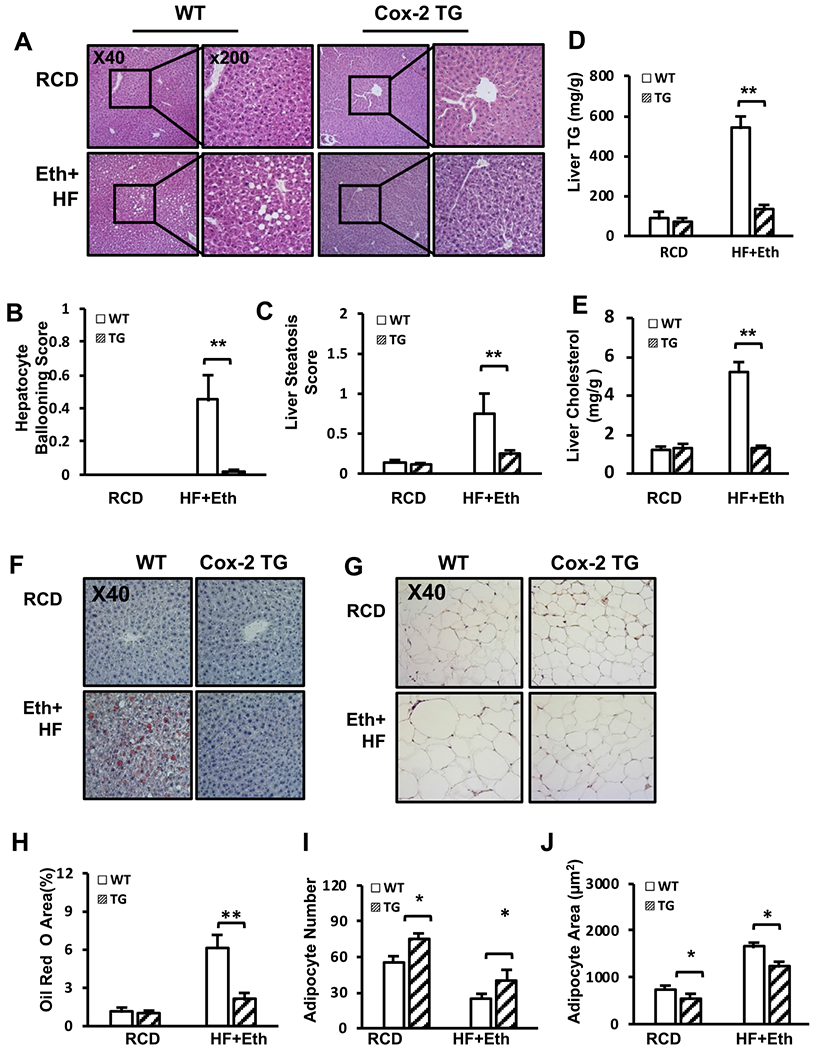
Cox-2 expression protects against HF+Eth-induced liver steatosis and adipose tissue ballooning. **(A)** Representative pictures of liver H&E staining from WT and TG mice on RCD or HF+Eth diet after 5 weeks. **(B–C).** Histological scores of hepatocyte ballooning and liver steatosis. **(D**—**E)** Levels of liver triglyceride and cholesterol after mice were fed with RCD or HF + Eth diet. **(F–G)** Representative pictures of liver Oil Red O and eWAT H&E staining from WT and TG mice on RCD or HF+Eth diet after 5 weeks. **(H)** Percentage of Oil Red O area. **(I–J)** Adipocyte number and adipocyte area in Fig. 3F. Each staining was performed on three successive sections per slide, and at least nine sections from three non-consecutive slides per mouse were examined. N = 6—8 mice for each group, data are presented as mean ± SD. *p < 0.05, **p < 0.01 WT vs. HF + Eth; #*p* < 0.05, ##*p* < 0.01 RCD vs. HF+Eth.

To further investigate how TG mice are protected from the hepatic injury resulting from the HF+Eth diet, we performed histological analyses with liver and adipose tissue of WT and TG mice on regular chow or HF+Eth diet. While the liver cellular structures of WT mice and TG mice were similar in mice on the regular control diet, HE staining of the liver showed HF+Eth-fed WT mice displayed moderate hepatic steatosis with mild ballooning, while hepatic COX-2 expression protected mice from HF + Eth diet-induced hepatocyte change (Fig. 3A—C). Oil Red O staining of livers from WT on HF + Eth diet exhibited a moderate amount of lipid drop deposition (Fig. 3E and 3G), which was consistent with increased liver triglycerol and cholesterol levels (Fig. 3F and H). There was no visible Oil Red O staining in the TG mice on the HF+Eth diet, and liver TG resembled the levels of WT and TG mice on the regular diet (Fig. 3D).

The liver is the crucial organ used to transport nutrients to other tissues, and excessive nutrition leads to white adipose tissue proliferation and hypertrophy. In addition to significant increased epigonadal white adipose tissue size (Fig. 3G), we also observed in WT mice adipose tissue hypertrophy and decreased adipocyte number compared to TG mice on the HF+Eth diet (Fig. 3G, H and I). In mice fed with the RCD, the average adipocyte number was increased with a decrease in adipocyte size compared to COX-2 TG mice (Fig. 3G, I and J). These indicate that lipid metabolism is already changed in COX-2 TG mice on the regular diet.

### Enhanced AKT insulin signaling-mediated lipogenesis in the livers of COX-2 TG mice with RCD/HF+Eth-induced metabolic change

Lipid metabolism is tightly orchestrated, with its net outcome being a balance of *de novo* lipogenesis, and lipid uptake vs. lipid utilization as β-oxidation, lipid secretion, and lipid autophagy. Fatty acid synthesis from glucose under insulin-Akt regulation and direct transport into the liver are two significant components for lipogenesis (Bechmann et al., 2012; Titchenell, Lazar, & Birnbaum, 2017). To further explore the underlying mechanism of how COX-2 protects the liver from HF diet-induced liver steatosis and insulin resistance, we investigated several critical nutritional components of liver metabolism, along with the main regulatory pathway of insulin-AKT response.

**Fig. 4.**
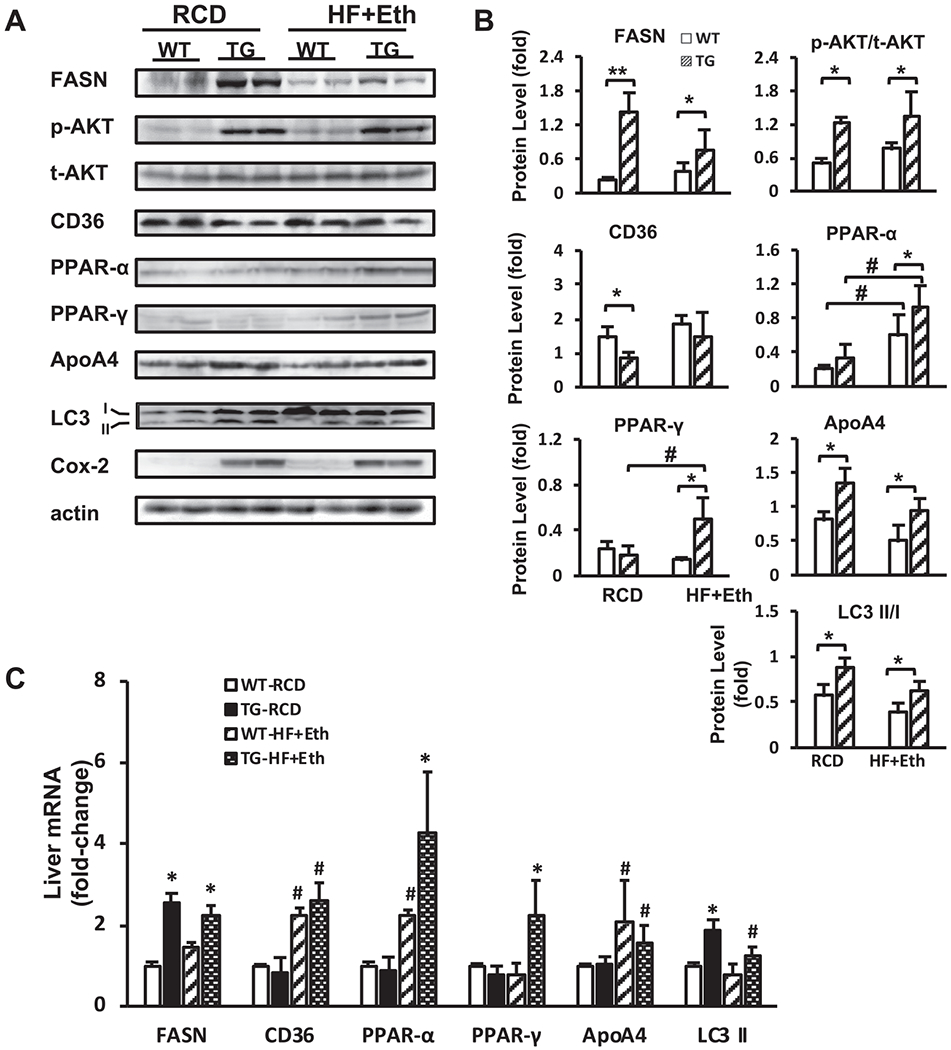
Enhanced lipogenesis and lipid expenditure were present in the livers of COX-2 TG mice and WT mice fed with RCD/HF+ diets. **(A)** Representative Western blot analysis of WT and COX-2 TG mice under RCD or HF+Eth diet. All mice were treated with 10 IU/kg insulin i.p. 15 minutes before euthanization. Antibody manufacture and dilution can be found in Table 2. **(B)** Ratio of densitometric analysis of Western blot data normalized using β-actin. p-AKT was normalized by total AKT. **(C)** Liver cytokine mRNA expression level of *Fasn, Cd36, PPAR-α, PPAR-γ*, Apoa4, and *LC3 II* genes in WT and TG mice on RCD or HF+Eth diet for 5 weeks. N = 6–8 mice in each group, data are presented as mean ± SD. *p < 0.05, **p < 0.01 WT vs. HF+Eth; #*p* < 0.05, ##*p* < 0.01 RCD vs. HF+Eth.

Although there was no significant difference in H&E presentation between WT and COX-2 TG mice on the regular control diet, Western blotting showed that several vital regulators in lipid metabolism were dramatically changed in TG mice. Fatty acid synthase (FASN) was upregulated in TG mice compared to WT mice on the regular diet, and the TG mice presented with an activated phospho-AKT pathway responding to insulin stimulation. Although HF+Eth diet attenuated this upregulation, it was still a significant increase compared to WT mice on the same diet. p-AKT/AKT was also high compared to being mildly upregulated in WT mice on the HF+Eth diet. Western blot showed that the scavenger receptor, CD36, was marginally inhibited in TG mice on the control diet compared to WT mice, but there was no difference between WT and TG mice fed the HF + Eth diet for 5 weeks (Fig. 4A and B).

Further, we analyzed the gene expression level of the above key components of nutritional metabolism regulation in the livers of WT and TG mice on control or HF+Eth diets. The primary regulators of these pathways indicated that the activated status of lipogenesis was consistent with elevations in their protein levels (Fig. 4C).

### Enhanced lipid disposal pathways were present in the livers of COX-2 TG WT mice on RCD/HF+Eth diets

The increased lipid synthesis observed in TG mice seemed to contradict the relative lack of lipid droplet accumulation (Fig. 3A and F) in the liver, prompting us to look at the “out” branch of lipid metabolism. We examined three major lipid dispenser pathways, including the TG secretion protein ApoA4, regulators of β-oxidation PPARa and PPARg, and autophagy markers LC3 I and LC2 II.

We found that lipid transport out of the liver through ApoA4 was upregulated in COX-2 TG mice on the control diet relative to WT mice. The HF+Eth diet inhibited ApoA4 expression in both WT and TG mice, but TG mice still displayed a higher ApoA4 level compared to WT mice. Energy expenditure through β-oxidation was also increased, notably with increased level of PPARα. Unexpectedly, PPARγ, a notable mediator in the oxidative stress pathway, was significantly upregulated in COX-2 TG mice on the HF+Eth diet compared to WT mice. The third lipid dispenser marker, LC3 II/I ratio, was escalated in COX-2 TG mice compared to WT mice on the control diet. This upregulation was attenuated in both TG and WT mice on the HF+Eth diet, but there was still a small but significant upregulation in TG mice compared to WT mice (Fig. 4A and B). Gene expression levels of the above key components of nutrition metabolism regulation in the liver were consistent with protein levels (Fig. 4C). Therefore, here we concluded that COX-2 TG mice displayed accelerated lipogenesis and lipid utilization, and the latter may account for the protective function to the stress of the high fat plus ethanol diet.

### Multiple acetylation and de-acetylation pathways were activated in the COX-2 TG-mediated nutritional reprogramming

Emerging evidence has suggested that histone and protein epigenetic modification is closely associated with the COX-2 pathway and nutritional rerouting (Francés et al., 2013, 2015; Motiño et al., 2016; Yu et al., 2007). Our study is the first, to our knowledge, to investigate the expression level of HAT and HDACs to detect any acetylation activity change in histone or cytosolic proteins in the context of hCOX-2 TG mice liver under nutritional stress. Furthermore, the upregulation of FASN expression in protein and mRNA levels in TG mice on regular and HF+Eth diets prompted us to explore expression levels of SNAIL1 and SIRT1, two relevant regulators that link protein/histone acetylation and energy metabolism (Liu et al., 2018; Rowe et al., 2011; Sekiya & Suzuki, 2011; Sun et al., 2016).

**Fig. 5.**
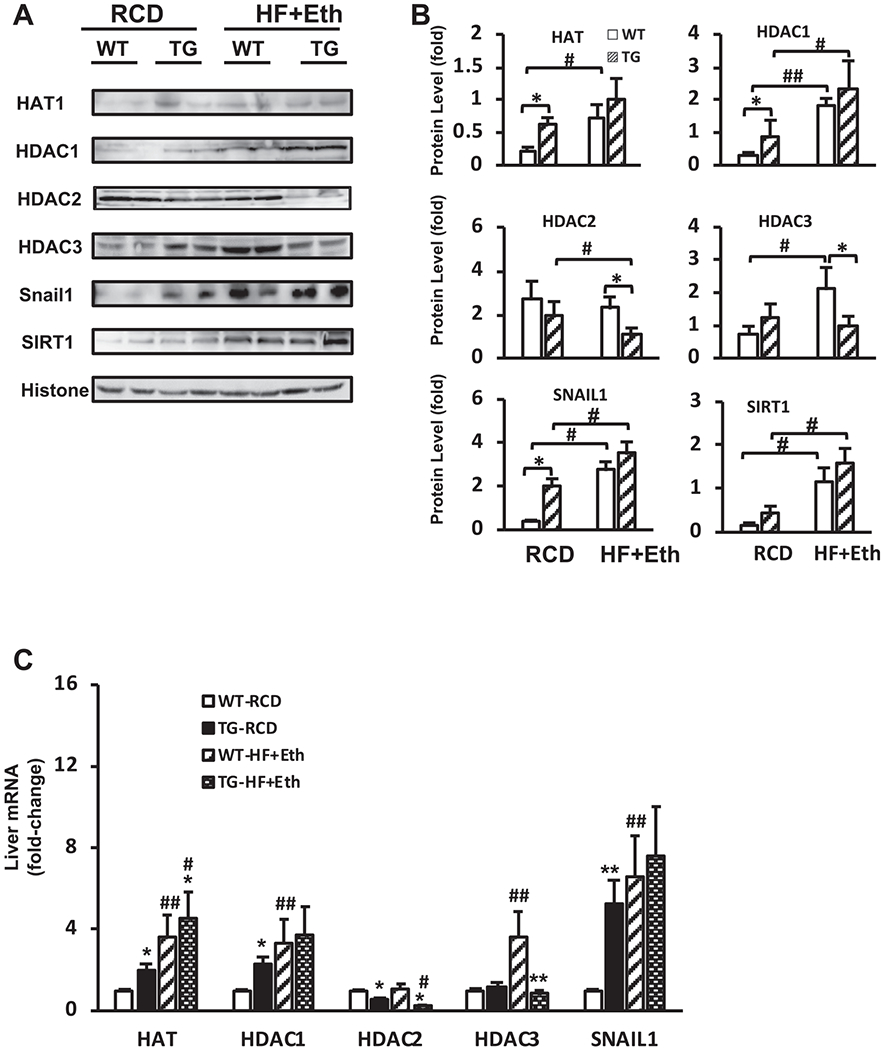
Multiple acetylation and de-acetylation pathways were activated in the COX-2 TG-mediated nutritional reprogramming. **(A)** Representative Western blot analysis of WT and COX-2 TG mice under RCD or HF+Eth diet. All the mice were treated with 10 IU/kg insulin i.p. 15 minutes before euthanization. Antibody manufacture and dilution can be found in Table 2. **(B)** Ratio of densitometric analysis of Western blot data normalized using histone. **(C)** Liver cytokine mRNA expression level of *HAT, HDAC1, HDAC2, HDAC3*, and *SNAIL1* in WT and TG mice on RCD or HF+Eth diet for 5 weeks. N = 6–8 mice in each group, data are presented as mean ± SD. *p < 0.05, **p < 0.01 WT vs. HF+Eth; #*p* < 0.05, ##*p* < 0.01 RCD vs. HF+Eth.

We observed that the expression level of histone acetyl-transferase (HAT1), the primary regulator of histone acetylation, was slightly upregulated in TG mice on the regular diet. It was also moderately upregulated in WT mice on the HF + Eth diet, and even more upregulated in TG mice on the HF+Eth diet (Fig. 5A). Since histone and protein acetylation includes de-acetylation processes as well, we also examined the expression level of *Hdac*1/2/3 and *Snail* 1 genes (Fig. 5A and B). In mice on the regular control diet, we detected a deficient level of HADC1 in WT mice, while it was moderately upregulated in COX-2 TG mice. The HF+Eth diet increased HDAC1 levels in WT mice also, but the most significant increase was observed in the COX-2 TG mice fed the HF+Eth diet. Conversely, HDAC2 expression was suppressed in the COX-2 TG mice compared to WT mice on the control diet. While the HF+Eth diet did not change the expression level of HDAC2 in WT mice, it was significantly reduced to an almost undetectable level in TG mice on the HF+Eth diet. We also explored the HDAC3 protein level in the liver because of its reported correlation with diabetes (Aroor et al., 2014). The HDAC3 level was already increased in COX-2 TG mice, but it was mostly upregulated in the WT mice on the HF+Eth diet. Of note, the expression level of HDAC3 was heavily attenuated in the group of TG mice on the HF+Eth diet.

As SNAIL1 collaborates with HDACs 1 and 2 to execute the de-acetylation of FASN in the insulin/SNAIL1 pathway, we also analyzed SNAIL1 protein level in the liver (Liu et al., 2018). SNAIL1 was increased in the COX-2 TG mice on the regular diet, as well as in WT mice on the HF + Eth diet; the HF+Eth diet further raised SNAIL1 expression (Fig. 5A and B).

In addition to the above three class I HDACs, we also investigated a class III HDAC SIRT1, which plays an essential role in nutrition metabolism (Cantó & Auwerx, 2012). SIRT1 stayed at a similar level in COX-2 TG mice on the regular control diet, but it was moderately upregulated in both WT and COX-2 TG mice on the HF + Eth diet, implying that SIRT1 is involved more in the diet-induced pathway than the inflammatory pathway that initiates energy rerouting.

Further, we examined the gene expression RNA levels of critical components of DNA/protein acetylation and de-acetylation regulation in the livers of WT and TG mice on control or HF+Eth diets. The primary regulators of DNA and protein acetylation and de-acetylation indicated that the activated status of adding or removing the acetyl group to the histone and cytosolic protein was consistent with mRNA level (Fig. 5A—C). Our studies indicate that COX-2 functions to reduce hepatic lipid accumulation by modifying the acetylation levels of an intricate complex of genes involved in nutritional regulation.

## Discussion

We have investigated nutritional metabolism in WT and COX-2 TG mice on RCD or HF+Eth diet for 5 weeks. Our results indicated that COX-2TG mice were protected from nutritional stress-induced obesity, insulin resistance, and liver damage. Mechanistically, hepatic COX-2 overexpression enhanced AKT insulin signaling and increased fatty acid synthesis, but its anti-lipogenic effect was mediated through β-oxidation and hepatic autophagy. Protein acetylation and de-acetylation were actively involved in the COX-2 TG mice compared to WT mice in the RCD and HF+Eth diets, reflecting the involvement of COX-2 in nutritional reprogramming.

In this study, we focused on the combined effect of the high fat and ethanol diet on the background of WT and COX-2 TG mice, based on the clinical significance of the coexistence of ethanol drinking and Western diet. In WT mice, the HF+Eth diet induced significant body weight gain, adipose tissue deposition, and hyperlipidemia, coupled with glucose intolerance and insulin resistance. This result is consistent with a recently published murine model using free access ethanol consumption with an HF diet, which also leads to body mass increase and adiposity, and glucose intolerance (Coker et al., 2020). Clinical and experimental animal results indicated that ethanol may increase the insulin sensitivity and alleviate the glucose intolerance (Coker et al., 2020; Feng et al., 2012; Gelineau et al., 2017; Hong, Smith, Harvey, & Núñnez, 2009; Traversy & Chaput, 2015). However, it is intriguing that the comorbidities of a high fat plus ethanol diet does not always synergistically promote hepatic lipid disposition and metabolic dysfunction, although high fat diet or ethanol diet alone could significantly impact glucose and lipid metabolism (Rehm et al., 2017; Steiner, Crowell, & Lang, 2015). Since our experiments did not include separate groups of HF or ethanol-only diet, our results in Figs. 1 and 2 suggest that ethanol does not totally mitigate the deteriorative effect of the HF diet in our 5-week diet regimen.

We found that hepatocyte COX-2 overexpression protected the mice from HF + Eth-induced fatty liver and metabolic dysfunction. COX-2 TG mice gained less weight, showed improved glucose tolerance, serum and hepatic lipid profiles, and less fatty liver damage (Figs. 1—3). It has been reported that COX-2 TG mice showed a similar protection on a high fat-alone diet (Francés et al., 2015), including less weight gain, less insulin resistance, and decreased hepatic steatosis, indicating that COX-2 TG has a profound effect on energy usage. Because the insulin-AKT pathway is a critical signaling pathway regulating multiple steps of energy usage, we speculate that COX-2 TG may protect mice from HF+Eth diet-induced metabolic dysfunction through enhancing the insulin-AKT signaling pathway. At the early stage of fasting, serum glucose level is maintained by the glycogenolysis from hepatic glycogen under tight regulation of the insulin-Akt pathway (Jensen, Kiersgaard, Sørensen, & Mikkelsen, 2013; Longo & Mattson, 2014). With prolonged starvation, the liver fully oxidizes fatty acids transported from adipose tissue to sustain glycemia (Jensen et al., 2013; Longo & Mattson, 2014). In COX-2 TG mice on the HF+Eth diet, we found enhanced AKT phosphorylation compared to WT mice when mice were stimulated by insulin (Fig. 4A and B). Moreover, COX-2 TG mice displayed default Akt phosphorylation even without insulin stimulation under the regular diet (Francés et al., 2015; and our unpublished data).

We also noticed that fasting (4 hours) plasma glucose in the ITT test was lower in TG mice compared to the WT mice on the regular control diet, indicating activation of the constitutive insulin-AKT pathway, or lower glycogen storage in the livers of COX-TG mice. Interestingly, the plasma glucose level in prolonged fasting (16 hours) in COX-2TG mice was compensated to a comparable level, probably by increasing fatty acid uptake of COX-2TG mice, as shown in Fig. 2A. The insulin-AKT pathway also links glucose regulation and fatty acid metabolism. Accordingly, our results indicate that on the normal chow diet, hepatic COX-2 overexpression increased FASN level in the liver as well (Fig. 4A), with an increase in p-AKT(S473) level. The effect of COX-2 on FASN was ameliorated in the HF+Eth diet. Interestingly, p-AKT(S473) stayed at the high level compared to the HF+Eth diet, suggesting an uncoupling of the AKT/FASN pathway under a high fat and alcohol diet.

PPARg’s role in adipogenesis and lipid metabolism is well established (Wang et al., 2017; Wang, Nakajima, Gonzalez, & Tanaka, 2020). In this study, we have shown that PPARγ was increased in the COX-2 TG mice under the HF+Eth diet. Our observation that COX-2 regulates glucose and fatty acid metabolism is supported by another study (Motiño et al., 2016). It has been reported that in a chronic high fat with binge alcohol consumption murine model, inflammation induced through PPARγ upregulation is an independent modulator in the progression toward liver steatosis (Wang et al., 2017). Activation of PPARγ through COX-2 TG may further upregulate the inflammation in the liver and shift the energy preservation to energy consumption. In all, our study suggests that energy metabolism is accelerated in multiple tissues in COX-2 TG mice. Additionally, it has been previously demonstrated that COX-2 TG mice exhibited increased energy expenditure by upregulating expression of genes that regulate thermogenesis in adipose tissue through enhanced PGE2 (Francés et al., 2015). It would be of great interest to explore how this hepatic-specific, pro-inflammatory gene in the transgenic mice could spare other deteriorative effects of acute inflammation while preserving this energy rerouting efficiency.

COX-2 is an inducible key enzyme that plays a critical role in orchestrating the inflammatory response (Francés et al., 2015). Our results indicated that the COX-2 level was not induced in the 5-week HF + Eth diet paradigm (Fig. 4A), and it could not be further upregulated in the COX-2TG mice under the HF+Eth diet. Contrary to knowledge that COX-2 could be readily regulated by proinflammatory stimuli like LPS *in vitro*, our result is consistent with the current understanding that high COX-2 expression is only detected in partial hepatectomy animal models (Font-Nieves et al., 2012; Martín-Sanz, Casado, & Boscá, 2017). However, we cannot exclude the possibility of a transient or low level of COX-2 expression in the adult liver under some extreme conditions. COX-2 protein level was detected in an acute high-dose insulin challenge mouse model in our lab (unpublished data), and several labs have shown that the chronic inflammation induced by the hepatitis virus was accompanied by COX-2 expression (Chen et al., 2015; Cheng et al., 2004). Those COX-2 expressions under rare pathophysiological conditions may lead to changes in chromatin structure and levels of cytosolic proteins to modulate nutritional rerouting. It has been well known that ethanol alters protein and histone acetylation (Wang, Gao, Zakhari, & Nagy, 2012). Among the various acetylated proteins, SIRT1 is a key player in crosstalk between inflammatory cytokine regulation and energy rerouting. It has also been shown to inhibit *de novo* lipogenesis and increase fatty acid *β*-oxidation (Ding, Bao, & Deng, 2017). In our study (Fig. 5A), SIRT1 is upregulated by the HF+Eth diet. COX-2 overexpression did not change the SIRT1 protein level under either diet, indicating that SIRT1 participates in the HF+Eth diet-induced liver steatosis, but did not contribute significantly to the protective function of COX-2 TG in HF+Eth-induced metabolic dysfunction. It has been reported that chromatin remodeling is involved in COX-2-induced cellular events, as evidenced by a recent study that demonstrates the DNA methylation of specific oncogenes in COX-2-overexpressing mice (Chen et al., 2017). While recent studies suggesting that histone and mitochondrial protein acetylation in a high fat diet evoke protein hyper-acetylation are controversial (Carrer et al., 2017; Choi & Friso, 2010; Meyer et al., 2018; Peterson et al., 2018), the contribution to the hyper-acetylation status from the ethanol diet is more widely accepted (Choi & Friso, 2010; Jung & Metzger, 2015; Kriss et al., 2018; Lin et al., 2018; Zhong et al., 2010; Zhong & Lemasters, 2018). A recent approach using ^13^C-labeled ethanol together with mass spectrometry in an ethanol binge drinking mouse model provided the quantitative evidence of histone hyper-acetylation status (Kriss et al., 2018), and we would like to employ this method in our future studies to significantly improve our understanding of the critical steps in nutritional rerouting in dietary stress. In all, these findings provide a crosssection of the acetylation of several vital proteins, reflecting an interesting role of protein epigenetic dysregulation under nutritional stress. Activation of the hepatic sirtuins 1-nuclear sterol regulatory element binding protein 1-histone H3 axis has been found to downregulate the expression of genes encoding lipogenic enzymes and to suppress the synthesis of hepatic fatty acids, favoring prevention of high saturated fat against alcoholic fatty liver (You, Cao, Liang, Ajmo, & Ness, 2008). Sirtuins 3 has been identified in the mitochondria as nicotinamide adenine dinucleotide-dependent deacetylase (Onyango, Celic, McCaffery, Boeke, & Feinberg, 2002; Schwer, North, Frye, Ott, & Verdin, 2002), which deacetylates and activates several mitochondrial fatty acid oxidation enzymes in the liver. SIRT3 expression and activity are highly dependent on prevailing dietary conditions, and both are down-regulated in response to excess nutrition of a high fat diet (Thapa et al., 2018). Our future studies will be carried out to determine whether alcohol consumption can affect sirtuin 3 expression and activity in the mitochondria and mitochondrial fatty acid oxidation enzymes.

In conclusion, our current study demonstrates that COX-2 TG mice are protected from high fat and ethanol-induced insulin resistance and metabolic disorder. Key epigenetic modifications found in our study may be related to the effect of COX-2TG mice and a diet high in fat and alcohol.

## Figures and Tables

**Table 1:** RT-PCR primer list.

Primers	Forward (5′-3′)	Reverse (5′-3′)
TNF-α	TTCCGAATTCACTGGAGCCTCGAA	TGCACCTCAGGGAAGAATCTGGAA
IL-iβ	ACGGACCCAAAAGATGAAG	TTCTCCACAGCCACAATGAG
IFN-γ	ATGAACGCTACACACTGCATC	CCATCCTTTTGCCAGTTCCTC
IL-6	ATCCAGTTGCCTTCTTGGGACTGA	TAAGCCTCCGACTTGTGAAGTGGT
FASN	TGCTCCCAGCTGCAGGC	GCCCGGTAGCTCTGGGTGTA
CD36	AATGGCACAGACGCAGCCT	GGTTGTCTGGATTCTGGA
PPAR-α	AGAGCCCCATCTGTCCTCTC	ACTGGTAGTCTGCAAAACCAAA
PPAR-γ	TATGGAGTGACATAGAGTGTGCT	CCACTTCAATCCACCCAGAAA
ApoA4	CCAATGTGGTGTGGGATTACTT	AGTGACATCCGTCTTCTGAAAC
LC3b	TTATAGAGCGATACAAGGGGGAG	CGCCGTCTGATTATCTTGATGAG
HAT1	AAGTGTAACACCAACACAGCA	CGAAAGCAGTTTCATCATCCCC
HDAC1	AGTCTGTTACTACTACGACGGG	TGAGCAGCAAATTGTGAGTCAT
HDAC2	GGAGGAGGCTACACAATCCG	TCTGGAGTGTTCTGGTTTGTCA
HDAC3	GCCAAGACCGTGGCGTATT	GTCCAGCTCCATAGTGGAAGT
SNAIL1	CACACGCTGCCTTGTGTCT	GGTCAGCAAAAGCACGGTT
GADPH	TGAACGGGAAGCTCACTGG	TCCACCACCCTGTTGCTGTA

**Table 2:** Antibody list.

Antibodies	Vendor	Catalog number	Dilution
FASN	CELL SIGNALLING	3180S	1:1000
CD36	Abcam	ab133625	1:500
PPAR-α	Proteintech	66826-1-Ig	1:500
PPAR-γ	Proteintech	16643-1-AP	1:500
APOA4	Proteintech	17996-1-AP	1:300
LC3	Proteintech	14600-1-AP	1:1000
AKT-S473	Proteintech	66444-1-Ig	1:500
T-AKT	Proteintech	60203-2-Ig	1:1000
COX-2	CELL SIGNALLING	12282T	1:1000
β-ACTIN	SIGMA	A-5316	1:3000
HDAC1	Proteintech	10197-1-AP	1:300
HDAC2	Proteintech	12922-3-AP	1:300
HDAC3	Proteintech	10255-1-AP	1:300
HAT1	Proteintech	11432-1-AP	1:300
SNAIL1	Proteintech	13099-1-AP	1:500
Sirt1/	Proteintech	13161-1-AP	1:500
Histone H3	Proteintech	17168-1-AP	1:1000
